# Dental amalgam teaching phase-out - a step too soon? Foundation trainees' experience of amalgam use in dental school compared to practice: a mixed-methods survey

**DOI:** 10.1038/s41415-023-6228-4

**Published:** 2023-09-08

**Authors:** Noor Jebur, Karen Vinall-Collier, Abdul-Ahad Umair, Vishal R. Aggarwal

**Affiliations:** 453168548342495594656 School of Dentistry, University of Leeds, United Kingdom; 567654694524823292967 School of Dentistry, University of Leeds, UK; Associate General Dental Practitioner, Bupa Dental Care, United Kingdom

## Abstract

**Aim** This study aimed to explore disparities in experience of UK dental foundation trainees in amalgam use at dental school compared to their first year in dental practice.

**Methods** A modified version of the 'survey of Yorkshire dentists' targeted the dental foundation trainees. A mixed-method of quantitative and thematic content analysis was undertaken for the close-ended and free-text responses, respectively.

**Results** Only 35% of participants described their confidence level as 'satisfactory' in placing dental amalgam before starting their dental foundation training and 51% scored the level of teaching between 'very poor' and 'satisfactory'. In total, 63% of respondents were in favour of receiving additional support in placing amalgam restorations during their undergraduate training. Participants indicated that the consequences of amalgam phase-out are increased appointment times and decreased quality of care due to patient and health service unaffordability of amalgam alternatives.

**Conclusion** These findings raise concerns regarding newly qualified undergraduates' confidence, experience and skill in placing amalgams. This suggests the need to increase undergraduate education and training to improve undergraduates' skills and confidence in amalgam procedures to reduce the gap between the dental schools and real-world practice.

**Supplementary Information:**

Zusatzmaterial online: Zu diesem Beitrag sind unter 10.1038/s41415-023-6228-4 für autorisierte Leser zusätzliche Dateien abrufbar.

## Key points

- Amalgam is a frequently used material by foundation dentists and is the material of choice for large posterior restorations.
- Foundation dentists feel ill-equipped with regards to confidence, experience and skill in placing amalgams.
- There is a need to increase undergraduate education and training in amalgam placement to reduce the gap between dental schools and real-world practice.

**Supplementary Information:**

Zusatzmaterial online: Zu diesem Beitrag sind unter 10.1038/s41415-023-6228-4 für autorisierte Leser zusätzliche Dateien abrufbar.

## Introduction

In response to The Minamata Convention in 2013, an international treaty focussing on challenging the environmental pollution of toxic materials, dental amalgam phase-out was agreed upon due to the hazardous effect of its mercury content on human health and the environment.^1^ The European Union agreed to amalgam phase-down and complete phase-out by 2030.^2^

Although there is a big push to phase out dental amalgam use, it is still one of the most widely used restorative materials and the backbone of dental services in most countries,^3^ including the UK, where dental practices, especially those funded by the NHS, rely on its regular use as a restorative material for posterior teeth.^4^^,^^5^ Quantitative analysis showed trends towards a slight reduction in the number of amalgam restorations used by the NHS between the financial years 2007-2017. However, it still represented a large proportion with amalgams, accounting for 42% of the restorations placed in the 2016-2017 financial year.^6^

The ease of placement, need for less moisture control, robustness, low cost and high strength make it the material of choice,^7^ and in comparison to its biggest rival - composite - it is, on average, $48 cheaper, with almost half the lifetime cost (the expense of replacing the same restoration if it had failed) and an additional lifetime of 36.9 months.^8^ With dental practices constrained by time and cost, it comes as no surprise why dental practitioners may prefer amalgam, especially when its alternatives generally come with the burden of higher costs and longer operating times.^9^

To mitigate the consequences of a complete phase-out, the FDI World Dental Federation, International Association for Dental, Oral and Craniofacial Research, British Dental Association and World Health Organisation issued policy statements that amalgam use should be continued where alternatives are 'sub-optimal' due to financial or clinical reasons.^10^^,^^11^

However, in response to the objectives set out by the Minamata Treaty,^1^dental schools have been urged to increase undergraduates' education about mercury-free restorative materials and support research and development of mercury-free dental materials. Based on this, there has been a worldwide increase in composite teaching compared with amalgam.^12^ The UK is following similar trends: three surveys of teaching conducted by Lynch *et al.* between the years 2006-2018 to evaluate the amount of teaching of posterior composite compared to that of amalgam across 18 dental schools in the UK and Ireland showed a drop in amalgam restorations placed by students from 70% to 33%, and an increase in the amount of composites resin restorations from 30% to 66%.^4^^,^^13^^,^^14^ However, despite such trends in education, dental amalgam is still widely used in clinical practice, especially for posterior restorations, and remains the go-to material for dental services in many countries, including NHS dental practitioners in the UK. Therefore, while dental schools are reducing the amount of teaching and training regarding amalgam restoration compared to composites, this raises concerns regarding dental graduates' preparedness for amalgam use.

Therefore, this study aimed to explore disparities in experience of UK dental foundation trainees (DFTs) in amalgam use at dental school compared to their first year in dental practice

## Materials and methods

### Study design

A questionnaire survey was used to assess and explore the experience of UK DFTs' experience and their confidence in dental amalgam restoration and their views on undergraduate teaching. The survey is a modified version of the 'survey of Yorkshire dentists' which was used in a recently published study: 'Assessing the perceived impact of post Minamata phase down on oral health inequalities: a mixed-methods investigation'.^6^ A section of the questionnaire investigated the current practice (images of a few cavity preparations were shown and participants were asked to choose what they would choose as a restoration material), confidence, views and time taken placing amalgam, alongside estimated figures on the amount of amalgam placed in practice per week. The survey was modified to match the desired target population and the purpose of the study, through consultation with relevant literature, input from experts in the field, and piloting with key stakeholders. A combination of close-ended (Likert-scale) and open-ended questions was used. Responses of the close-ended questions were quantitatively analysed and thematic content analysis was carried out for the open-ended free-text responses.

Full details of the questionnaire and survey are available in the online Supplementary Information.

The study was approved by the school of Dentistry Students' Ethics Committee (DSEC - reference FYP2020Amalgam).

### Piloting

The questionnaire was piloted by distributing to ten DFTs. Informal feedback was obtained regarding areas for improvement. The pilot study found that the questionnaire was able to be completed within an appropriate timeframe and asked the right questions to target the aims of the study.

### Sampling and recruitment

We used the Yamane formula to calculate our sample size.^15^ We needed 57 subjects to provide an 80% power and 95% confidence in detecting differences between amalgam use in dental school versus practice.

Participants were recruited through different methods, including snowball sampling via social media platforms, such as relevant dental Facebook and WhatsApp groups of DFTs and newly qualified dentists. Convenience sampling was also used by asking peers who knew DFTs that were suitable to take part in the study. The online link to complete the questionnaire was sent to participants via these channels and included a participant information sheet. Participants were then free to complete the questionnaire if they so wished and no identifiable information was collected on the questionnaire so we did not have personal details of participants completing the online questionnaire. By completing the questionnaire, participants consented to take part and were free to withdraw by not completing the questionnaire.

### Inclusion and exclusion criteria

Participants included were dentists currently working as DFTs in the UK or who completed their foundation training within 12 months, and working a minimum of 17 hours per week. We excluded any graduates from non-UK universities or those who have not carried out a dental foundation training programme.

## Results

In total, 84 responses were received from the online survey, which exceeded our required sample of 57.

### Choice of restorative materials

**Fig. 1:**
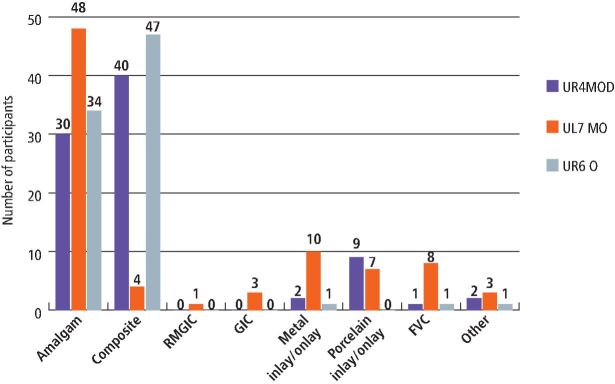
Choice of restorative material for different cavity preps assuming the patient has no preference and is happy for the clinician to choose

When respondents were shown images of different cavity preparations and asked about their choice of restorative material, amalgam was frequently chosen, especially in posterior teeth and more complicated restorations that involve more than one surface, as seen in Figure 1. A staggering 57% of respondents chose amalgam to be their restorative material of choice for a mesio-occlusal cavity, while only 4.8% chose composite for the same tooth. When it came to a premolar, however, nearly 48% of participants chose composite for a mesio-occlusal distal cavity while only 36% of respondents chose amalgam.

### Factors influence the choice of the restorative material

**Fig. 2:**
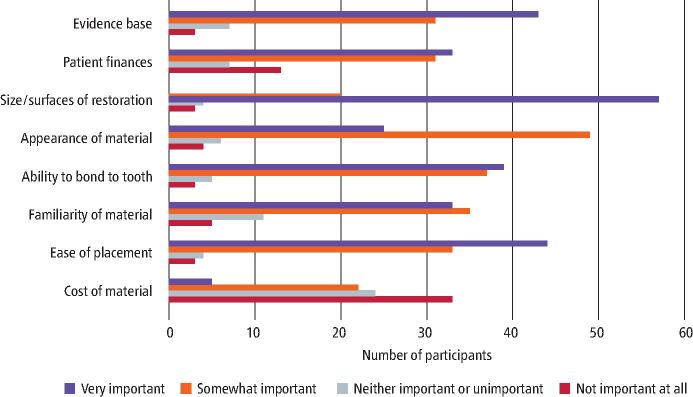
Importance of different factors influencing choice of restorative material

The size/surfaces of the restoration and ease of placement were the most important factor according to the respondents, while the cost of the dental materials was generally the least important factor to consider when choosing the restorative material, as seen in Figure 2.

### Frequency in amalgam placement

**Fig. 3:**
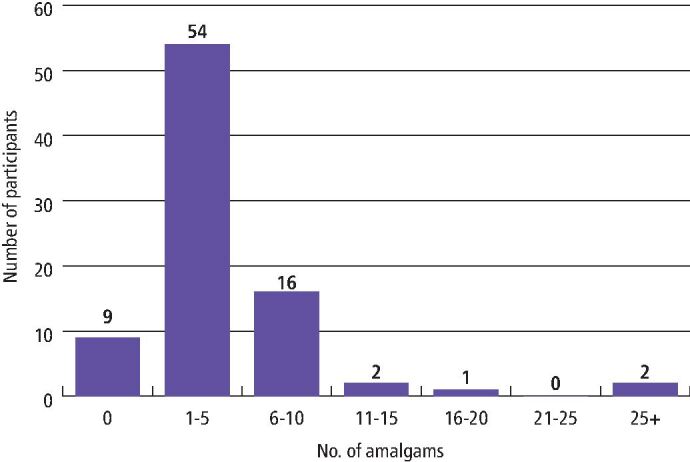
Average number of amalgam restorations placed in practice per week

In total, 75 participants were placing at least one amalgam per week, 54 were placing on average 1-5 amalgam a week, 16 were placing 6-10 and two were managing 25+ within the week, as shown in Figure 3.

### Confidence levels and time taken in placing amalgam

**Fig. 4:**
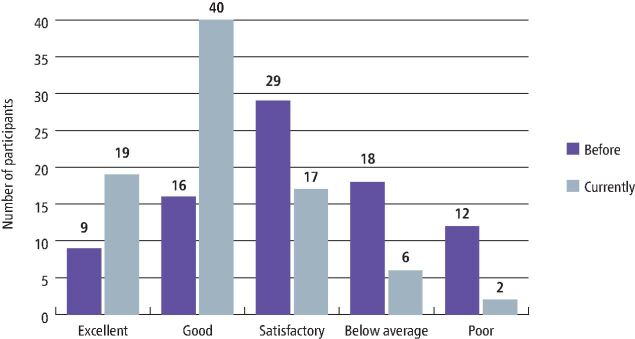
Level of confidence in placing amalgam as a restorative material before starting foundation training compared to completing foundation training/currently

Most of the participants (35%) chose 'satisfactory' when describing their confidence level in placing dental amalgam before starting their dental foundation training. However, when asked about their current confidence levels, 48% of participants chose their confidence level as 'good'. Only 23% said their current confidence level was 'excellent' while only 11% chose 'excellent' before their training (Fig. 4).

**Fig. 5:**
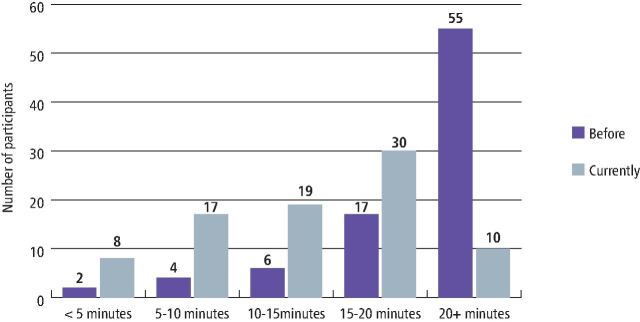
Average time taken to solely place an amalgam restoration into a cavity prepped first molar before starting foundation training compared to completing foundation training/currently

The majority of DFTs (65%) said that before their foundation training, their average time to place an amalgam within an existing cavity in a first molar tooth was 20 minutes or above. There was a slight improvement as the majority of DFTs (36%) selected 15-20 minutes when answering about their current average time (Fig. 5).

More than half of the participants (51%) scored the level of teaching between very poor and satisfactory. Around 63% of respondents were in favour of receiving additional support when asked if they feel it would be beneficial to them to have some form of additional support in placing amalgam restorations during their undergraduate training.

### Thematic content analysis of open-ended questions

#### Concerns over alternative materials

When participants were questioned whether they have any concerns over the alternative materials to amalgam, the majority (79.8%) of respondents answered 'no'. For the participants who answered 'yes', the general theme was based on concerns about issues in gaining moisture control with resin-based materials. Furthermore, a select number of respondents had concerns over the greater amount of time required to place the composites over amalgam and the need for aerosol generating procedures for the finishing of composite.

#### If amalgam was no longer an option

Participants reported their perceptions with free-text responses (n = 84). The main themes identified where the respondents indicated it would affect practice are discussed below. A summary of the themes is presented in Table 1.

**Table 1:** Summary of content analysis of open-ended questions

Higher level theme	Coding theme	N	Quotes	
Increased appointment times	Increased appointment times	24	'Need longer appointment times as need rubber dam for composite'	'A lot more time consuming if only composite was available but GIC is a lot quicker but a lot weaker'
Decreased quality of care	Difficulty and failure when poor moisture control	17	'Limits tx options for cavities where moisture control is not feasible'	'More failed restortions when poor moisture control'
	Poorer quality of restoration GIC	10	'I would have to use GIC in areas where I cannot obtain sufficient moisture control, leading to decreased quality of restorations as GIC is not as suitable'	'GIC not strong enough'
	Less restorable teeth	5	'I believe it would make some teeth (that could be saved with an amalgam restoration) unrestorable'	'Would render more teeth "unrestorable" or have poor prognosis inappropriate resin restorations'
	Complex restoration will no longer be treated by the NHS	3	'Would be difficult for large posterior fillings on the NHS'	'Would make deep restorations on the NHS almost impossible'
Affect treatment decision-making	More indirect restorations would be required	4	'It would probably mean more indirect restorations where I was unable to achieve good moisture control to place a composite as I wouldn't feel confident with GIC for large restorations long term'	'It would probably mean that some teeth would move through the restorative cycle faster (need root canal treatment + crown/extraction under local anaesthetic where previously they could've had an amalgam before needing these treatments)'
	Cost	9	'Most won't be able to afford'	'It would be more expensive'
	NHS guidelines	3	'The reason I use amalgam is because it's the material of choice for posterior fillings on NHS due to time/cost. Would use composite everyday but not feasible under NHS'	'Composite not available on NHS if occlusal surface included, patient budget affected. GIC not strong enough'
	Few alternatives to amalgam	3	'If subgingival and moisture control isn't optimal there are few alternatives to amalgam'	'Limited restorative materials'
Don't use amalgam	Practice only allow composite	2	'Would not affect my practice as I never use amalgam'	'My practice doesn't allow amalgam. Composites only'
	Preference for composite	2	'Not really, use composite mostly followed by GIC'	'I would just use composite'

The theme with greatest consensus in response was that if amalgam was no longer an option, this would increase appointment time (28.5%; n = 24). This theme correlates strongly with others and was mentioned most often by participants in relation to moisture control and time taken for placement and finishing of alternative materials. Decrease in quality of care was the second most prominent theme from the responses as participants described the consequences of the lack of moisture control, which may lead to restoration failure and the need for more complex indirect restorations. The use of unsuitable materials, such as glass ionemer cement (GIC), and ultimately greater incidence of less restorable teeth was also mentioned as a concern. Participants described how more complex indirect restorations will no longer meet requirements for NHS treatment and such indirect restorations would be more destructive, costly and technique-sensitive if sub-gingival. In contrast, some participants (3.5%; n = 3) reported that amalgam phase-out would not affect them as they no longer used it.

## Discussion

The findings of our survey showed that amalgam is still a widely used dental material, especially for large posterior restorations. The respondents also reported a difference in confidence level regarding amalgam restoration before and after dental foundation training, whereby half the participants rated the level of dental amalgam undergraduate teaching to be unsatisfactory, and two-thirds in favour of additional support for amalgam placement. These findings raise concerns regarding newly qualified undergraduates' confidence, experience and skill in placing amalgams and are corroborated by previous research, with UK dental students reporting having gained more experience in placing posterior composites compared to dental amalgam,^16^ and having less confidence in amalgam use.^17^ Trends in amalgam teaching in UK dental schools show a gradual reduction in amalgam teaching when compared to composites.^4^^,^^13^^,^^14^ This indicates a disparity and disconnect between teaching and clinical practice, which might result in creating dentists with reduced clinical experience and confidence in the placement of amalgam restorations.

It is important to try to reduce this gap in confidence levels by increasing the amount of undergraduate amalgam training in dental school clinics, including amalgam placement on phantom heads within clinical skills labs, as the majority of the participants reported placing at least 1-5 amalgam on average per week post qualification. It is clear that amalgam is a frequently used material even among novice dental practitioners, especially in the NHS, and this has also been confirmed by previous research.^5^^,^^6^^,^^18^^,^^19^

When respondents were shown images of cavity preparations and asked to choose from a list of materials, amalgam was frequently chosen, alongside composite. A total of 48 respondents chose amalgam as their material of choice for a mesio-occlusal cavity while only four picked composites. Choice of dental material by participants depended on ease of use, cost and size/surfaces of the restoration.^7^^,^^20^ This was confirmed by the theme in our content analysis, which showed that appointment times would considerably increase if amalgam was no longer an option, and this correlates strongly with other themes related to moisture control and time taken for placement, polishing and finishing of alternative materials.

The unavailability of amalgam was perceived by participants as having a direct impact on quality of care, with participants describing the consequences of poor moisture control for subgingival restorations leading to restoration failures, which in turn would lead to need for more complex and costly indirect restorations. Participants reported that the indirect effect of removing amalgam as a restoration may lead to the use of less suitable materials, such as GIC, and ultimately, greater incidence of extraction of teeth that are deemed unrestorable using alternatives to amalgam. This would lead to widening of oral health inequalities, as alternative treatments would be unaffordable by patients from deprived populations.^6^

### Limitations of the study

More information regarding the geographical location of where respondents carried out their dental foundation training would have been useful. In total, 33 respondents claimed patient finances to be very important when deciding upon restorative materials, 31 respondents stated it was somewhat important, and 13 agreed it was not important at all. Many DFTs work in NHS practices that serve areas of deprivation, so it would have been useful to find out whether they are placing more amalgams than those training in more affluent areas, and whether they have a different approach based on patient affordability when deciding upon restorative materials.

### Future research

The findings of the current study raise concerns over the preparedness of dental undergraduates in placement of dental amalgams post qualification. However, we only explored this from the perspective of DFTs. Future research needs to explore views of foundation trainers and dental school educators. Our previous qualitative study in this area^6^ showed that dental school educators reported a reduction in amalgam teaching, which is confirmed by our current findings. Anecdotal evidence suggests that foundation trainers are having to train DFTs on amalgam use. Further qualitative research involving foundation trainers, dental educators and DFTs is needed to explore the extent of this lack of preparedness and its implications on patients and clinical staff who support DFTs in practice.

## Conclusion

Our findings suggest that dental schools need to be aware that amalgams are used more frequently by graduates than perhaps anticipated and that they perceive themselves to be under-prepared in the use and placement of amalgam restorations. The clinical and educational impact of this understanding is that undergraduate dental curricula should take steps to improve the knowledge and practical skills of dental students in placing amalgams to better prepare them for real-word practice and ensure safe practice.

## Supplementary Information

Supplementary Information (PDF 303KB)
