# Supplementary material for: Dental amalgam teaching phase-out - a step too soon? Foundation trainees' experience of amalgam use in dental school compared to practice: a mixed-methods survey
Source: Br Dent J. 2023 Sep 8;235(5):329–34. doi: 10.1038/s41415-023-6228-4 (PMC10491489; doi:10.1038/s41415-023-6228-4)
Supplement: Supplementary file 1 — Supplementary Information (PDF 303KB) [file 41415_2023_6228_MOESM1_ESM.pdf]

Exploring dental foundation trainees' experience of amalgam as a restoration material in dental school compared to within practice

INFORMATION SHEET

We would like to invite you to take part in a survey exploring your experiences with amalgam

Why we are doing this survey?

We would like to hear about your thoughts and experiences in dealing with amalgam as a restorative material and whether these have changed after doing your dental foundation training in NHS practices. We want to find out whether there is a significant discrepancy between the level of preparation dental students feel they are given through clinical teaching of amalgam and the amount of amalgam they find themselves using in the clinical settings of their dental foundation training.

Why am I being asked to take part?

You have been asked to take part as you are either in the midst of completing your dental foundation training or you have completed your training within the last 3 months. The survey will be sent to 283 DFTs – males and females; of different ages, from different universities and working in different practices around the UK. By taking part it will help us understand whether this significant discrepancy exists and if so whether any action should be taken by dental schools to close this gap in teaching.

What will happen to me if I take part?

If you agree to take part, you will be given the online survey to fill out. There will be no need to sign a consent form if you're willing to take part. If at any point you feel that you want to discuss the project with us we will provide details for you to do this.

Confidentiality will be achieved by anonymising all data to ensure it cannot be traced back to you. No personal questions of any sort will be asked and there will be no direct access to the your email address either. Furthermore, Jisc operates an

information Security Management System (ISMS) to ensure all data from respondents remains secure on the database.

Will I be paid for taking part?

The survey is anonymous therefore we won't be able to contact participants for payment.

Do I have to take part?

It is completely your choice whether or not to take part, you can withdraw from the study at any time. If you agree to take part but during the survey you simply decide that you no longer wish to go on then you drop out at any time. However, if you decide after submitting the survey that you want to withdraw, this will not be possible as all information submitted by participants is anonymous.

What will happen to the information collected at the group?

If you consent to take part, everything will be kept confidential and handled strictly in accordance with the consent that you have given and also the 1998 Data Protection Act.

What will happen to the findings of this study?

The study will take up to February 2021 to complete. When it is completed a report will be written based on the findings. You can request to have a copy of this report for yourself if you'd like. We aim to publish our findings in professional and academic journals by the end of this study.

Who is organising the research?

The survey was organised by myself (Abdul-Ahad Umair – a fifth year dental student) and my two supervisors (Dr Vishal Aggarwal and Karen Vinall Collier).

Who has reviewed the study?

The study has been reviewed by The University of Leeds Dental Research Ethics Committee (DSEC) to protect your rights and safety.

What do I do if I have concerns?

You can send us a message on the email address attached at the bottom of this sheet.

How to contact us for further information?

Thank you for taking the time to read this information. If you choose to take part you will be given a copy of this information sheet and your signed consent form to keep

If you want further information about the survey, please contact:

Abdul-Ahad Umair, ll15aau@leeds.ac.uk alternatively you can contact the supervisors in charge of this research study: Dr Vishal Aggarwal, V.R.K.Aggarwal@leeds.ac.uk or Karen Vinall Collier, K.A.Vinall@leeds.ac.uk

Thank you for taking the time out to read this information and if you do decide to take part, your involvement will be much appreciated.

Questionnaire for Survey of Dental Foundation Trainees

Question 1:
On average, how many hours of clinical duties do you work per week?
☐ < 8
☐ 9 – 16
☐ 17 – 24
☐ 25 – 32
☐ 33 – 40
☐ 41 – 48
☐ 48 +

Question 2:
How many amalgam restorations do you currently place per week on average?

☐ 0
☐ 1-5
☐ 5-10
☐ 11-15
☐ 16-20
☐ 21-25
☐ 26+

Question 3:
Looking at the following factors, how important are they in your choice of restorative material?

	Not important at all	Neither important or unimportant	Somewhat important	Very important
Cost of material				
Ease of placement				
Familiarity of material				
Ability to bond to tooth				
Appearance				
Size/surfaces of restoration				
Patient finances				
Evidence base				

Question 4:
In your normal practice what would you restore this tooth with? Please rank 1-3 in order of your personal preference, the patient has no preference and is happy for you to advise what you consider to be the best restoration.

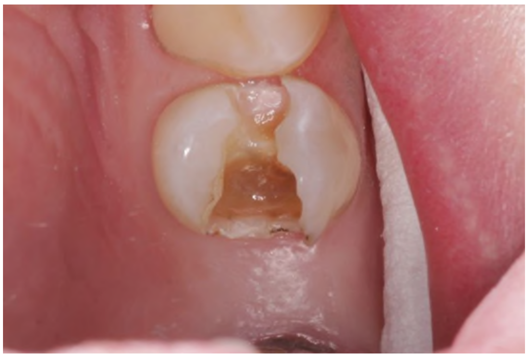

UR4 MOD
- ☐ Amalgam
- ☐ Composite
- ☐ Resin-modified GIC
- ☐ GIC
- ☐ Metal inlay/onlay
- ☐ Porcelain inlay/onlay
- ☐ Full coverage crown
- ☐ Other – ……………………………

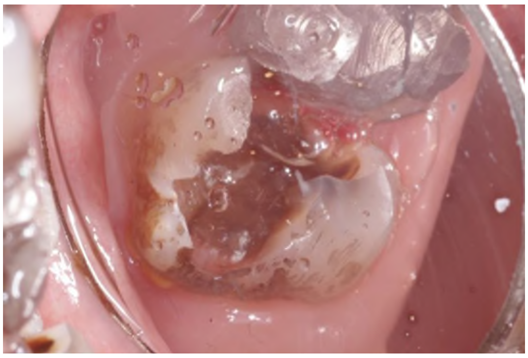

UL7 MO (extends subgingivally)
- ☐ Amalgam
- ☐ Composite
- ☐ Resin-modified GIC
- ☐ GIC
- ☐ Metal inlay/onlay
- ☐ Porcelain inlay/onlay
- ☐ Full coverage crown
- ☐ Other – …………………………

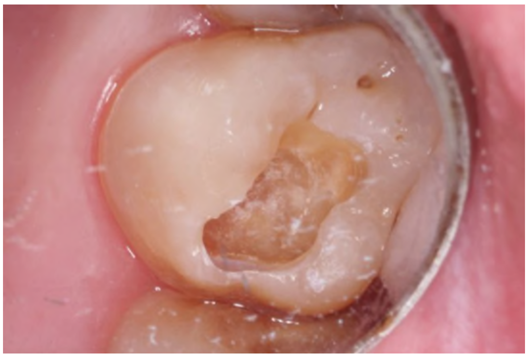

UR6 O - large
- ☐ Amalgam
- ☐ Composite
- ☐ Resin-modified GIC
- ☐ GIC
- ☐ Metal inlay/onlay
- ☐ Porcelain inlay/onlay
- ☐ Full coverage crown
- ☐ Other – ………………………

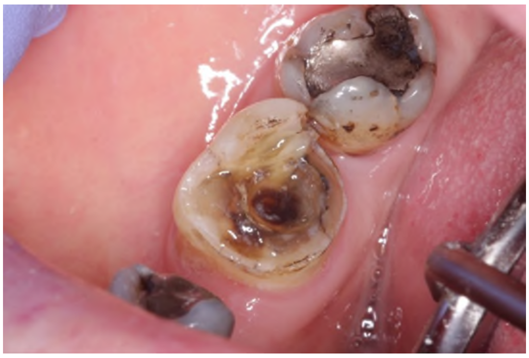

LR6 - MODL
- ☐ Amalgam
- ☐ Composite
- ☐ Resin-modified GIC
- ☐ GIC
- ☐ Metal inlay/onlay
- ☐ Porcelain inlay/onlay
- ☐ Full coverage crown
- ☐ Other – ………………………..

Question 5:
How would you score the level of clinical teaching of amalgam at your university after doing your foundation training on a scale of 1 – 5? 1 being very poor and 5 excellent.

- ❑ 1 – very poor
- ❑ 2
- ❑ 3

- ☐ 4
- ☐ 5 – excellent

Question 6:
How would you score the level of clinical teaching of amalgam at your university after doing your foundation training on a scale of 1 – 5? 1 being very poor and 5 excellent.

- ☐ 1 – very poor
- ☐ 2
- ☐ 3
- ☐ 4
- ☐ 5 - excellent

Question 7:

How would you score your confidence in placing amalgam restorations before starting your foundation year training?

- ☐ excellent
- ☐ good
- ☐ satisfactory
- ☐ below average
- ☐ poor

Question 8:

How would you score your confidence in placing amalgam restorations now that you are doing your foundation training/have completed your foundation training?

- ❑ excellent
- ❑ good
- ❑ satisfactory
- ❑ below average
- ❑ poor

Question 9:

On average how long did it take you to complete a MOD amalgam restoration for a first or second molar before starting your foundation year training?

- ❑ < 5 minutes
- ❑ 5 - 10 minutes
- ❑ 10 - 15 minutes
- ❑ 15 - 20minutes
- ❑ 20+ minutes

Question 10:

On average how long does it take you to complete a MOD amalgam restoration for a first or second molar now that you are doing your foundation training/have completed your foundation training?

- ❑ < 5 minutes
- ❑ 5 - 10 minutes
- ❑ 10 - 15 minutes
- ❑ 15 - 20 minutes
- ❑ 20+ minutes

Question 11:
When considering alternative restorative materials to amalgam, do you have any concerns over the materials that could be used?

☐ Yes ☐ No

If Yes then please explain why below:

Question 12:
a) If you were not able to use amalgam as a material how would this affect your practice?

b) Do you think that amalgam is still a useful material for restoring posterior teeth?

☐ Yes ☐ No ☐ Unsure

Please explain your answer below:

If you have any further comments that you would like to make on this subject then please note them below.

Thank you for taking the time to complete this questionnaire.
